# Quantitative assessment of eye movements using a binocular paradigm: comparison among amblyopic, recovered amblyopic and normal children

**DOI:** 10.1186/s12886-022-02579-5

**Published:** 2022-09-09

**Authors:** Yulian Zhou, Hewei Bian, Xiaobin Yu, Wen Wen, Chen Zhao

**Affiliations:** 1 Department of Ophthalmology and Vision Science, Eye & ENT Hospital, Shanghai Medical School, Fudan University, Shanghai, 200031 China; 2 Key Laboratory of Myopia, Ministry of Health, Fudan University, Shanghai, 200031 China; 3 Shanghai Key Laboratory of Visual Impairment and Restoration, Fudan University, Shanghai, 200031 China; 4 State Key Laboratory of Medical Neurobiology and MOE Frontiers Center for Brain Science, Institutes of Brain Science, Fudan University, Shanghai, 200032 China; 5 Beijing Institute of Ophthalmology, Beijing Tongren Eye Center, Beijing Tongren Hospital, Capital Medical University, Beijing, 100730 China; 6 Department of Ophthalmology and Vision Science, Eye & ENT Hospital, Shanghai Medical School, Fudan University, 83 Fenyang Rd, Shanghai, 200031 China

**Keywords:** Eye movement, Fixation, Saccade, Amblyopia, Binocular viewing

## Abstract

**Background:**

To investigate the eye movement functions in children with amblyopia and recovered amblyopia by a binocular eye-tracking paradigm.

**Methods:**

Eye movements of 135 pediatric subjects (age range: 4–14 years), including 45 amblyopic children, 45 recovered amblyopic children and 45 age-similar normal controls, were recorded under binocular viewing with corrected refractive errors (if any). The deviation of gaze positions relative to the target location was recorded as the mean from both eyes. Main outcome measures included fixation deviations (degree) along horizontal and vertical axes in the sustained fixation test (Fix-X, Fix-Y) and visually guided saccade test (Sac-X, Sac-Y), which were compared across the three groups and between each two groups.

**Results:**

All the four deviations were significantly larger in the amblyopia group compared to the other two groups, indicating increased inaccuracy of sustained and post-saccadic fixations in amblyopia. However, there was no significant difference in deviations between recovered amblyopic children and normal controls. Repeated measures showed similar results overall and within each group. Mild to moderate amblyopes and severe amblyopes did not differ in the four deviations. No significant interaction was found between subject groups and clinical characteristics (age, refractive status, and anisometropia).

**Conclusion:**

Amblyopic children have poor eye movement functions with increased inaccuracy of sustained and post-saccadic fixations, which appear to be restored in children with recovered amblyopia. Binocular assessment of eye movements provides valuable indicators of functional recovery in amblyopia.

**Supplementary Information:**

The online version contains supplementary material available at 10.1186/s12886-022-02579-5.

## Background

Fixations and saccades are two important functions of voluntary eye movements for visual perception of objects and scenes in humans. Fixation is an important function to hold the central visual field on a target for a certain period of time, which is critical to obtain sufficient details of visual targets [1]. Saccades are fast eye movements to reorient gaze, changing the image of an interested object from one to another onto the fovea, which are important for everyday activities such as improving the accuracy and precision of hand reaching [2] and manual manipulation [3], and the efficiency of reading [4]. Fixations and saccades with accurate eye positioning provide humans with continuous information from the outside world.

Amblyopia is diminished vision due to inadequate visual experience during infancy and early childhood without structural eye abnormalities [5]. Abnormal eye movements in patients with amblyopia have been reported, such as increased frequency and inaccuracy of saccades in reading [4], increased fixation instability [4, 6–9], and prolonged latency and decreased precision in saccades [10]. The paradigms used in previous studies above were typically conducted in monocular status, while the eye movement deficits of amblyopia patients under binocular viewing, similar to daily life, have been rarely reported. Only a handful of studies evaluated eye movements under binocular viewing, but analyzed the amblyopic eye (AE) or the fellow eye (FE) separately [4, 9, 10] and reported monocular outcomes rather than integrated data of both eyes. However, binocular integrated data, the mean of fixation deviation from both eyes in this study, could better simulate how binocular summation [11] effects on eye movement performance in real world. In addition, it remains unrevealed whether effective amblyopia intervention in childhood could additionally restore eye movement functions.

In this study, we described a binocular eye-tracking paradigm to assess the accuracy of sustained and post-saccadic fixations in three groups of subjects, including amblyopic, recovered amblyopic and normal children. The aims were to investigate whether amblyopic children had impaired eye movement functions, and whether timely and effective amblyopia intervention in childhood would simultaneously improve the functions.

## Methods

### Subjects

This study included 135 pediatric subjects aged 4–14 years who presented to the Department of Ophthalmology at Eye & ENT Hospital of Fudan University from Jan 2021 to Jan 2022. Subjects were assigned into three groups according to their clinical diagnosis, including the amblyopia group (*n* = 45), recovered amblyopia group (*n* = 45), and control group (*n* = 45). All the subjects were Chinese. The study adhered to the tenets of the Declaration of Helsinki and was approved by the institutional review board of the Eye & ENT Hospital of Fudan University. Written informed consent was obtained from the parents/guardians of the subjects and assent was obtained from children ≥ 7 years of age prior to their participation.

All the subjects underwent a comprehensive eye examination, including visual acuity with the Standard Logarithm Visual Acuity Chart (arithmetic scaled high-contrast E optotype; the only type of chart available to us at the clinic), refractive errors with a cycloplegic refraction (1% cyclopentolate), ocular alignment with a simultaneous prism cover test and a prism and alternate cover test, anterior segment examination with the slit lamp, stereopsis with the Titmus Stereo Test (Stereo Optical Co, Inc), fundus examination, and eye movement functions (before cycloplegia) with the binocular paradigm proposed in this study. For data analysis, refractive errors were converted to spherical equivalent (SE), the sum of the spherical power and half of the cylindrical power. Best-corrected visual acuity (BCVA) was converted to logarithm of the minimal angle of resolution (logMAR) and approximate Snellen equivalent was provided. Anisometropia was defined as an interocular difference in SE of 1.00 diopters [D] or more. Refractive error was defined by the SE in the more ametropic eye (myopia < -0.50 D; emmetropia within ± 0.50 D; hyperopia >  + 0.50 D).

Subjects were included only if they had no history of ocular trauma and/or ocular pathology (e.g., nystagmus, cataract, ptosis), no systemic disease (by-self report), no history of intraocular surgery, no measurable strabismus (≤ 5 PD at 6 m and 33 cm fixation with/without spectacle correction), and sufficient cooperation with the examinations. The inclusion criteria of each group were as follows:

#### The amblyopia group

Diagnosis of amblyopia at the most recent visit or unrecovered amblyopia with treatment less than 1 year, with BCVA of 20/30 or worse (20/50 for age 4 years; 20/40 for age 4 to ≤ 5 years) in the worse eye or an interocular difference in BCVA of two lines or more (≥ 0.2 logMAR) (according to the Amblyopia “PPP” guideline, 2017 [12]). Amblyopia associated with deprivation or uncorrected strabismus (> 5 PD at distance and/or near fixation) was excluded. BCVA in AE was used to classify the severity of amblyopia into mild to moderate (20/32–20/80) and severe (20/100 or worse) amblyopia.

#### The recovered amblyopia group

A history of amblyopia, with resolved visual acuity at the most recent visit after amblyopia treatment.

#### The control group

Normal or corrected-to-normal visual acuity in both eyes, stereoacuity ≥ 60 arc seconds, refractive errors within ± 6.00D sphere and ± 1.00D cylinder, absence or presence of anisometropia, and no history of amblyopia or other ocular diseases.

### Eye movement assessment

#### Apparatus

The experiment took place in a quiet and private room with a natural and constant luminance. A 32-inch 3-dimension (3D) monitor (resolution 1920 × 1080 pixels at a refresh frequency of 120 Hz; LG Electronics, Seoul, Korea) was used to present stimuli at a viewing distance of 80 cm. Subjects were asked to wear 3D polarized glasses with spectacle correction (if any) and seated on a non-wheeled but height-adjustable chair with the eyes at the same level as the screen center. Gaze positions were recorded with a 120 Hz remote eye tracker (Tobii Eye Tracker 5). The presentation of stimuli was generated by MATLAB (MathWorks, Natick, MA).

#### Calibration

The subjects were briefly familiarized with the procedure by the experimenter, and were asked to adjust and maintain the head position until the eye tracker could catch his/her both eyes optimally. A 3-point (X, Y = 0°, + 13.5°; -13.5°, -13.5°; + 13.5°, -13.5°; presented for 4 s at each location) calibration and validation of the eye tracker was run at the beginning of the main experiment and whenever necessary during the experiment. The subjects were asked to fixate their gaze on the calibration stimulus, a bright blue dot on a black background which was dynamically shrinking from a normal size (diameter 0.3°), and the binocular data was collected at the moment the dot disappeared. The subjects did not have to keep the head completely still during calibration as long as their eyes remained focused on the stimulus, since the eye tracker was able to track and correct for head movements simultaneously. The gaze positions were measured as separate horizontal and vertical components by the eye tracker, simultaneously and respectively for both eyes. Blinks or partial blinks were automatically detected and removed from analysis. The following main experiment would be initiated when calibration and its subsequent validation were acceptable with adequate accuracy for each eye.

#### Sustained fixation test

**Fig. 1:**
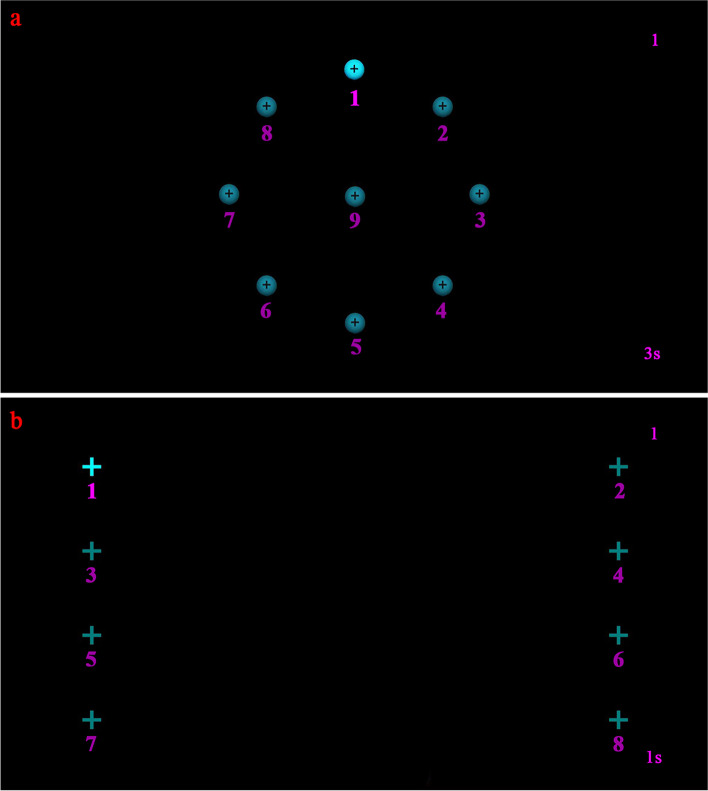
Schematic diagrams of the paradigm for eye movement assessment. During the test, there was always only one target presented on the screen, changing locations in a specified order. **a** Sustained fixation test. The target was presented in order from location 1 to 9, remaining for 3 s at each location. The purplish-red number “1” at the upper right corner was the cumulative number of gaze positions extracted from all samples grabbed at each location, recorded in real time from “1” to “5”. The “3 s” at the lower right was the actual sampling time for each location; **b** Visually guided saccade test. The target was presented in order from location 1 to 8, staying for 3 s at each location. Likewise, the purplish-red number “1” at the upper right corner was recorded in real time from “1” to “10” at each location. The “1 s” at the lower right was the actual sampling time for each location

The sustained fixation test measured the deviation of sustained fixations under static binocular-viewing condition. The subjects were instructed to fixate their gaze on a target on a black background, which was a bright blue dot (1.4° diameter) with a black cross-shaped center. The target appeared in a fixed order at 9 locations, 8 locations on a peripherical circle (8.3° radius) and 1 location in the center (Fig. 1a). It remained for 3 s on each location and automatically switched to the next location; however, data recording at each location was manually started by the experimenter until ensuring that the subject’s fixation had changed and sustained on the target. Fixation was defined by an oculomotor behavior shown under the effort to maintain the gaze in a predefined region [13]. Gaze positions after the manual start of data recording were considered fixations (the initiation phase of fixation [14] was therefore removed from analysis). At each target location, the eye tracker cumulatively extracted 5 samples from all gaze positions it grabbed. The mean horizontal and vertical deviations of these 5 gaze positions relative to the target location were calculated for each eye individually and recorded as the binocular mean values.

#### Visually guided saccade test

The visually guided saccade test measured the deviation of post-saccadic fixations under dynamic binocular-viewing condition. A bright blue cross-shaped target (1.4° diameter) appeared at 8 locations on a black background in turn, ± 17.8° horizontally alternately and + 8.3°, + 2.8°, -2.8°, -8.3° vertically sequentially, requiring a wide-range ocular motion (saccade) and succeeding fixation (Fig. 1b). The target was presented for 3 s at each location and then switched to the next location, with data recording started automatically. The horizontal and vertical gaze positions were measured as the subject attempted to fixate the gaze on the target after saccades. Considering saccade latency and fixation initiation [14] during the fixation alternations, the eye tracker only grabbed gaze positions from 800 to 1800 ms and extracted 10 samples at each location. Likewise, at each target location, the mean horizontal and vertical deviations of these 10 gaze positions relative to the target location were calculated for each eye individually and recorded as the binocular mean values.

Subjects were asked to repeat the experiment after 20 min to validate the stability of this method and to rule out potential learning effects. In addition, since the results were displayed on the screen immediately at the end of each test, a re-test was required once the results seemed unreliable due to the subject’s distraction during the test.

### Statistical analysis

The horizontal and vertical deviations (°) in the two tests were calculated for each subject by the equations below.

$$Horizontal\ deviation=\frac{1}{k}\sum_{1\le j\le k} \frac{1}{\mathrm{n}}\sum_{1\le i\le n}\frac{\left(\Delta {\mathrm{X}}_{Left}+\Delta {\mathrm{X}}_{Right}\right)}{2}$$

$$Vertical\ deviation=\frac{1}{k}\sum_{1\le j\le k} \frac{1}{\mathrm{n}}\sum_{1\le i\le n}\frac{\left(\Delta {\mathrm{Y}}_{Left}+\Delta {\mathrm{Y}}_{Right}\right)}{2}$$

In the equations, the parameter *n* was the number of gaze positions extracted at each target location, and the parameter *k* was the number of predefined locations where the target would appear during the test. As described above, *n* and *k* were 5 and 9 in the sustained fixation test, and 10 and 8 in the visually guided saccade test, respectively. ΔX and ΔY represented the horizontal and vertical deviations of each gaze position extracted relative to the target location, with subscripts *Left* and *Right* representing the left and right eye. The mean horizontal and vertical deviations from all target locations in each test were finally calculated as the main outcome measures.

For simplicity, the horizontal and vertical deviation were abbreviated as Fix-X and Fix-Y in the sustained fixation test, and Sac-X and Sac-Y in the visually guided saccade test, respectively. Small fixation deviations in the tests indicated accurate and controllable eye movements.

Normally distributed continuous data were presented as means with standard deviations (SD). Abnormally distributed continuous data were presented as median (25th percentile [P25], 75th percentile [P75]). Categorical variables were described as frequency counts and proportions (%). Group characteristics were compared using one-way analysis of variance (ANOVA) for normal-distributed continuous variables, nonparametric Kruskal–Wallis test for abnormal-distributed continuous variables, and Pearson’s χ^2^ test or Fisher’s exact test for categorical variables.

Since deviations were distributed right-skewed, Kruskal–Wallis test was used to compare the four deviations across the three groups, and pairwise comparisons were performed using Dunn test with the Bonferroni correction for multiple comparisons. Wilcoxon signed-rank test was used to compare repeated measures of deviations. Scheirer-Ray-Hare test, the non-parametric equivalent of ANOVA, was used to detect the interaction between subject groups and other clinical characteristics (age, refractive status, and anisometropia). Spearman’s rank correlation and Mann–Whitney U test was used for associations between clinical characteristics and deviations in the amblyopia group. *P* values less than 0.05 (two-sided) were considered statistically significant. Analyses were performed using the open-source statistical software R version 4.1.3 (R Foundation).

## Results

### Group characteristics

Clinical and demographic characteristics of the three groups were showed in Table 1. Groups did not differ in age (F_2,132_ = 1.79, *P* = 0.17) and sex (χ^2^ = 1.49, *P* = 0.47). BCVA in AE and FE in amblyopic children was respectively worse than that in the right eye (OD) and left eye (OS) in controls (Dunn, *Z* = 8.72, *P*_adj_ < 0.001; *Z* = 2.88, *P*_adj_ = 0.012), and also worse than that in previous AE and FE in recovered amblyopic children (*Z* = 7.51, *P*_adj_ < 0.001; *Z* = 2.57, *P*_adj_ = 0.030); while BCVA in recovered amblyopic children were similar with that in controls (Dunn, *P*_adj_ > 0.05). SE in OD and OS in controls was respectively different from that in AE and FE in amblyopic children (*Z* = 5.78, *P*_adj_ < 0.001; *Z* = 3.64, *P*_adj_ = 0.001), and also different from that in previous AE and FE in recovered amblyopic children (*Z* = 3.53, *P*_adj_ = 0.001; *Z* = 5.29, *P*_adj_ < 0.001); while SE in amblyopic children were similar with that in recovered amblyopic children (Dunn, *P*_adj_ > 0.05).

**Table 1:** Clinical and demographic characteristics in the three groups

	**Amblyopia**	**Recovered Amblyopia**	**Control**	***P* value**
No	45	45	45	NA
Age, Mean (SD), year	6.67 (2.31)	7.56 (2.45)	7.40 (2.40)	0.172 ^b^
Sex: Female, No. (%)	23 (51.1)	23 (51.1)	28 (62.2)	0.473 ^a^
Amblyopia severity ^c^, No. (%)				
No (20/16—20/25)	NA	45 (100)	45 (100)	NA
Mild to moderate (20/32—20/80)	36 (80.0)	NA	NA	
Severe (20/100—20/400)	9 (20.0)	NA	NA	
BCVA in AE/OD ^d^, Mean (SD), logMAR	0.37 (0.30)	0.04 (0.06)	0.02 (0.04)	< 0.001 ^e^
BCVA in FE/OS ^f^, Mean (SD), logMAR	0.08 (0.12)	0.03 (0.06)	0.02 (0.04)	0.007 ^e^
SE in AE/OD ^d^, Mean (SD), D	2.64 (3.63)	2.20 (2.71)	-0.50 (1.97)	< 0.001 ^e^
SE in FE/OS ^f^, Mean (SD), D	1.19 (2.64)	1.46 (2.04)	-0.29 (1.90)	< 0.001 ^e^
SE Refractive group ^g^, No. (%), D				< 0.001^a^
< -6.00	2 (4.4)	0 (0)	0 (0)	
-6.00 ~ -3.00	1 (2.2)	2 (4.4)	4 (8.9)	
-3.00 ~ -0.50	5 (11.1)	4 (8.9)	17 (37.8)	
-0.50 ~ + 0.50	0 (0)	3 (6.7)	11 (24.4)	
+ 0.50 ~ + 3.00	13 (28.9)	20 (44.4)	9 (20.0)	
+ 3.00 ~ + 6.00	21 (46.7)	13 (28.9)	4 (8.9)	
> + 6.00	3 (6.7)	3 (6.7)	0 (0)	
Anisometropia, No. (%)	30 (67.7)	23 (51.1)	15 (33.3)	0.007 ^a^

*F* Female, *BCVA* Best-corrected visual acuity, *logMAR* logarithm of the minimum angle of resolution, *SE* Spherical equivalent, *AE* The amblyopic eye, *OD* The right eye, *FE* The fellow eye, *OS* The left eye, *D* Diopter, *NA* Not applicable

^a^Pearson’s χ2 test or Fisher’s exact test

^b^one-way ANOVA

^c^The distance visual acuity is measured by Standard Logarithm Visual Acuity Chart, with approximate Snellen equivalent provided in parentheses. The visual acuity cutoff between “no amblyopia” and “mild to moderate amblyopia” was actually determined by the subject’s age according to Amblyopia “PPP” (2017) [12]

^d^BCVA in (previous) AE in the (recovered) amblyopia group, BCVA in OD in the control group

^e^Kruskal-Wallis test

^f^BCVA in (previous) FE in the (recovered) amblyopia group, BCVA in OS in the control group

^g^SE in the more ametropic eye (SE with larger absolute value) was used to define the refractive groups

### Main outcome measures

Main outcome measures included four deviations (°), including Fix-X and Fix-Y in the sustained fixation test, as well as Sac-X and Sac-Y in the visually guided saccade test. The four deviations were positively correlated with each other (Spearman correlation, *P* < 0.05), both overall and within each group. Data for the four deviations were presented as median (P25, P75).

In the control group, the four deviations were Fix-X 1.58 (0.84, 2.60)°, Fix-Y 2.18 (1.56, 2.61)°, Sac-X 3.28 (1.79, 4.19)° and Sac-Y 2.48 (1.71, 3.65)°, respectively. Between controls with and without anisometropia, who were similar in age (F_1,43_ = 3.61, *P* = 0.064) and sex (χ^2^ = 3.03, *P* = 0.082), no significant difference was found in Fix-X (Mann–Whitney, *U* = 179.5, *Z* = -1.096, *P* = 0.273), Fix-Y (*U* = 190.5, *Z* = -0.831, *P* = 0.406), Sac-X (*U* = 190.5, *Z* = -0.831, *P* = 0.406) and Sac-Y (*U* = 195.5, *Z* = -0.710, *P* = 0.477).

In the amblyopia group, the four deviations were Fix-X 2.58 (1.40, 5.30)°, Fix-Y 3.10 (2.19, 4.50)°, Sac-X 7.35 (4.93, 10.01)° and Sac-Y 4.45 (3.15, 5.85)°, respectively. Compared to the control group, amblyopic children had significantly larger Fix-X (Dunn, *Z* = 3.35, *P*_adj_ = 0.002) and Fix-Y (*Z* = 4.21, *P*_adj_ < 0.001) in the sustained fixation test, as well as significantly larger Sac-X (*Z* = 6.19, *P*_adj_ < 0.001) and Sac-Y (*Z* = 4.92, *P*_adj_ < 0.001) in the visually guided saccade test, indicating the eye movement deficits in amblyopia, with increased inaccuracy in sustained and post-saccadic fixations along both the horizontal and vertical axes.

In the recovered amblyopia group, the four deviations were Fix-X 1.45 (0.83, 2.96)°, Fix-Y 1.93 (1.53, 3.20)°, Sac-X 3.78 (2.38, 5.93)° and Sac-Y 2.98 (2.11, 4.96)°, respectively. Compared to the amblyopia group, children with recovered amblyopia had significantly smaller Fix-X (Dunn, *Z* = 3.06, *P*_adj_ = 0.007) and Fix-Y (*Z* = 3.78, *P*_adj_ < 0.001) in the sustained fixation test, as well as significantly smaller Sac-X (*Z* = 4.60, *P*_adj_ < 0.001) and Sac-Y (*Z* = 2.99, *P*_adj_ = 0.008) in the visually guided saccade test. However, compared to normal controls, children with recovered amblyopia had similar outcomes in both tests (Fix-X, *Z* = 0.29, *P*_adj_ = 1.000; Fix-Y, *Z* = 0.43, *P*_adj_ = 1.000; Sac-X, *Z* = 1.60, *P*_adj_ = 0.332; Sac-Y, *Z* = 1.93, *P*_adj_ = 0.159), indicating that the accuracy and controllability of sustained and post-saccadic fixations appeared to be greatly improved in children with recovered amblyopia.

**Fig. 2:**
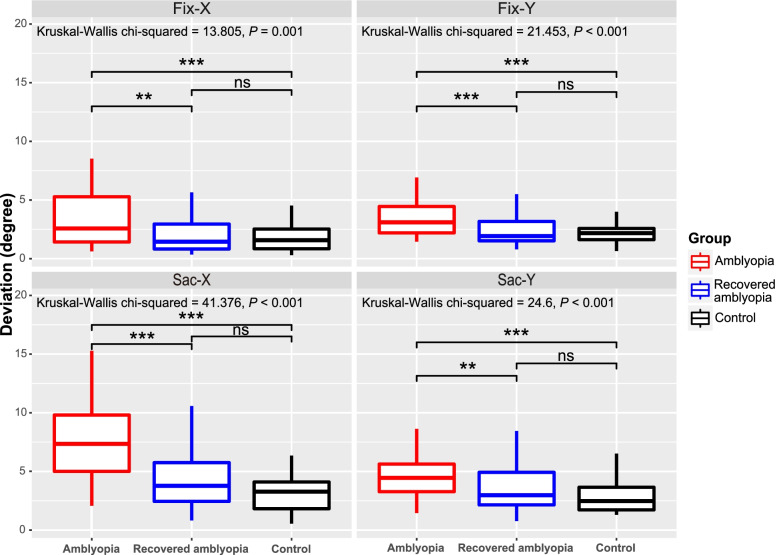
Comparison of the main outcome measures across groups. Fix-X/ Fix-Y: deviation (°) along horizontal/vertical axis in the sustained fixation test; Sac-X/ Sac-Y: deviation (°) along horizontal/vertical axis in the visually guided saccade test. Kruskal–Wallis test was used for comparison across the three groups, and Dunn test was used for pairwise comparisons (with the Bonferroni correction for multiple comparisons). ^***^ and.^**^ was significant difference between groups at *P* value < 0.001 and < 0.01, respectively; ns, *P* value > 0.05

**Fig. 3:**
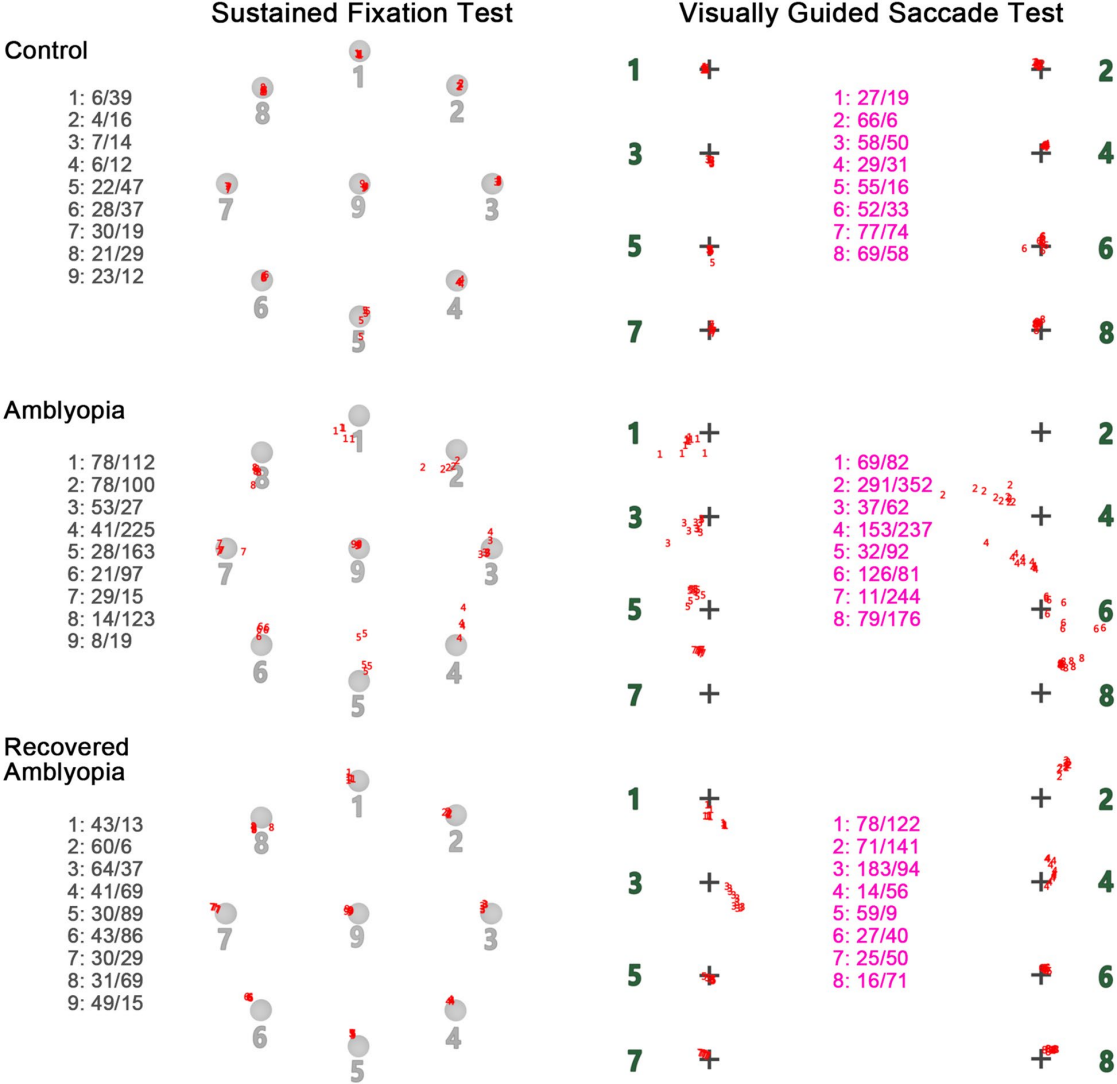
Examples of test results from the three groups. Left column: results of the sustained fixation test. On the left of each graph, the mean horizontal/vertical deviations (pixels) from both eyes were calculated respectively for 9 locations. On the right of each graph, the red numbers around the center of each location were the gaze positions extracted during the test. Right column: results of the visually guided saccade test. In the middle of each graph, the mean horizontal/vertical deviations (pixels) from both eyes were calculated respectively for 8 locations. On the two sides of each graph, the red numbers around the center of each location were the gaze positions extracted during the test. The amblyopic participant showed poor accuracy and precision in static and post-saccadic fixation, while the participant with recovered amblyopia showed similar results with the control

Each deviation was significantly different across the three groups (Kruskal–Wallis, *P* < 0.05) (Fig. 2). According to multiple comparisons, all the four deviations were significantly larger in the amblyopia group than the other two groups, while there was no significant difference in deviations between the recovered amblyopia group and the control group (Fig. 2). The eye movement deficits in amblyopic children could be visualized directly from the results displayed on the screen at the end of each test, showing large distance between gaze positions and the target location as well as large discreteness within gaze positions at each location, namely poor accuracy and precision in sustained and post-saccadic fixations, which seemed to be largely improved in children with recovered amblyopia (Fig. 3).

A total of 102 subjects (41 controls, 26 amblyopia, and 35 recovered amblyopia) had complete data of repeated measures. The four deviations measured from the first and second experiment were similar across all 102 subjects and within each group (see eTable 1. in Additional file 1). No significant interaction was found between subject groups and the clinical characteristics of age, refractive status and the presence of anisometropia (see eTable 2 in Additional file 2; the subgroup analysis was not pre-specified).

### Correlations between deviations and clinical characteristics in amblyopia

**Fig. 4:**
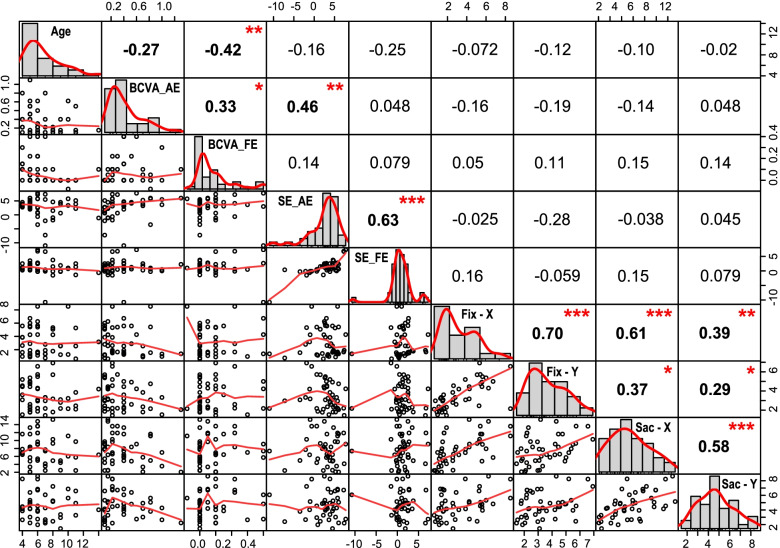
Correlations between deviations and clinical characteristics in the amblyopia group. The distribution plots of each variable were showed on the diagonal. The bivariate scatterplots with fitted lines were showed on the lower left panel. The correlation coefficients (Spearman) with significant levels as stars were showed on the upper right panel. ^***^, ^**^, ^*^ was significant correlation at *P* value < 0.001, < 0.01, < 0.05, respectively. Variables included age (year), BCVA_AE/FE (best-corrected visual acuity in the amblyopic/fellow eye, logMAR), SE_AE/FE (spherical equivalent of refractive error in the amblyopic/fellow eye, D), Fix-X/Fix-Y (horizontal/vertical deviation in the sustained fixation test, °), and Sac-X/Sac-Y (horizontal/vertical deviation in the visually guided saccade test, °). No significant correlation was found between deviations and the clinical characteristics

In amblyopic subjects, no significant correlation was found between the four deviations and clinical characteristics, including age, BCVA in AE/FE, and SE in AE/FE (Fig. 4). Male and female subjects did not differ in deviations (Mann–Whitney, *P* > 0.05). There was no significant difference in deviations between amblyopic children aged 4–7 years and 8–14 years (Mann–Whitney, *P* > 0.05). Amblyopic children with and without anisometropia significantly differed in Fix-X (*U* = 140, *Z* = -2.05, *P* = 0.041), whereas not in the other three deviations (Mann–Whitney, *P* > 0.05). Amblyopic children with myopia and hyperopia did not differ in deviations (Mann–Whitney, *P* > 0.05; the number of amblyopic subjects with emmetropia was zero), and there was also no difference in deviations among different SE refractive groups defined in Table 1 (Kruskal–Wallis, *P* > 0.05). No significant difference in deviations was found between mild to moderate amblyopes and severe amblyopes (Mann–Whitney, *P* > 0.05).

## Discussion

Amblyopia is traditionally considered as a monocular disease, however, over the past few decades, it has been increasingly appreciated as a binocular disorder caused by decorrelation of binocular stimulation or discordant visual experience during visual development [5]. Children with amblyopia have not only impaired monocular visual acuity, but also affected binocularity [15], eye-hand coordination [3], as well as eye movements both in the amblyopic eye and fellow eye [16]. Eye movement abnormalities in amblyopia have been reported in previous studies, but were typically assessed for each eye individually, under monocular viewing by the amblyopic eye or the fellow eye [4, 6–10]. However, we should focus on binocular skill deficits in amblyopia rather than in monocular status, since eye movement tasks are habitually performed with both eyes open in daily life. Several studies were conducted under binocular viewing condition, but evaluated each eye separately [4, 9, 10]. In the present study, we quantitatively assessed eye movement functions in amblyopic children using a binocular eye-tracking diagram. Deviations of sustained and post-saccadic fixations relative to the target were measured as the mean from both eyes. We found that amblyopic children have poor eye movement functions with increased inaccuracy of sustained and post-saccadic fixations compared to normal subjects, which was consistent with the general finding of a correlation between amblyopia and abnormal eye movements [4, 6–10].

In addition to amblyopic children, our study also included a group of children with recovered amblyopia to explore whether binocular eye movement deficits would be restored with resolved visual acuity after amblyopia intervention in childhood. In the clinic, amblyopia treatment is commonly terminated once normal visual acuity achieved, though binocular deficits persist in almost half of those with recovered visual acuity, including binocular perception eye position, interocular suppression, and stereoacuity [15, 17–20]. However, few studies evaluated recovery of eye movement functions in those patients. In the present study, we found that early and efficient intervention for amblyopia could additionally restore eye movement functions, since there was no significant difference in fixation deviations between children with recovered amblyopia and normal subjects. Quantitative assessment of eye movements might serve as an additional indicator of the efficacy of amblyopia treatment in clinical practice, providing a more comprehensive evaluation of visual function recovery. It might also become a tool for planning amblyopia management, especially in considering whether to continue treatment when changes of visual acuity were unsatisfactory.

Most of subjects (63.7%) in the present dataset were hyperopic. Visual blur due to uncorrected refractive error during fixation experiments might affect fixation properties [14, 21]. During sustained fixation, amplitude and rate of microsaccades increased with increasing refractive errors [21]. However, no significant interaction was found between subject groups and refractive status in this study. In amblyopic children, fixation deviations were found similar between myopes and hyperopes, and were independent of refractive errors as well. A possible reason was that in this study, the experiment was performed under corrected vision to avoid the influence of measurement errors under naked-eye viewing on the fixation accuracy [21]. However, it might obscure the effect of refractive errors at the same time, since a study found an increase in the amplitude of microsaccades under viewing with uncorrected refraction compared to corrected refraction [14]. In addition, the findings should be interpreted with caution since the number of amblyopic children with emmetropia was zero, which may affect the statistical power of interaction detection. Specially, sustained fixation might never be established in highly ametropic subjects with refractive errors of 5 D or more, which might affect their visual performance in addition to visual blur due to uncorrected refractive errors [21]. However, the effect of high ametropia on eye movements could not be analyzed based on the present dataset, since the number of highly ametropic subjects was quite small and unbalanced among groups.

Binocular decorrelation caused by anisometropia could disrupt visual development, leading to abnormal visual functions in children including eye movement abnormalities [6]. In our study, no significant interaction was found between subject groups and the presence of anisometropia, with 1.00 D SE as a cutoff. No significant difference in deviations was found between controls with and without anisometropia. Between amblyopic children with and without anisometropia, significant difference was only found in one of the four deviations (Fix-X). In addition to the potential effect of refractive correction during the experiment, this might also result from the limited and unbalanced samples in this study.

There was no significant interaction between subject groups and age strata (4–7 years, 8–14 years). Due to the complex interaction between amblyopia and visual development, as well as the varying interval from abnormal visual experience to the initiation of treatment, there might be variance of visual functions among amblyopic children of the same age. This again raises the necessity for assessment visual functions in addition to visual acuity throughout the amblyopia management.

Fixation deficits have been reported previously to be associated with severity of amblyopia [7, 22]. In our study, fixation deviations were not significantly correlated to visual acuity in amblyopic children. Mild to moderate amblyopes and severe amblyopes did not differ in the four deviations as well. The absence of correlation between deviations and amblyopia depth might be explained by following reasons. In this study, fixation deviations were recorded as binocular mean values rather than monocular data, and hence binocular summation [11] could have a potential effect on the outcome measures. Besides, different types of visual stimulus may have a different effect on measurements [23–25]. In addition, the sample size in each subgroup of amblyopia severity was limited, especially in the severe amblyopia subgroup.

In addition to sample size shortage and unbalanced subgroups, there are other limitations in our study. First, the outcome measures could quantitatively assess the accuracy of fixations, whereas the precision or stability of fixations could only be subjectively judged from the distribution of gaze positions on the screen, which may prevent comprehensive assessment and efficient follow-up of eye movement functions in amblyopia. Further design should measure both parameters indicating accuracy and precision of fixations to validate the findings of this study. Second, the fixation duration on each target location was set as 3 s in this study, which was confirmed sufficiently long for accurate and stable fixation in previous studies [21, 26] and would be a practical option in the clinical setting. However, previous work has suggested a strong dependence of fixation stability on fixation duration [27]. Further studies performing the experiment with increased fixation durations would help to investigate the effect of fixation duration on the outcome measures. In addition, patients with marked strabismus were not applicable for the tests, since quite different fixation coordinates would be provided by their left and right eye due to severe misalignment of the visual axes, and the eye tracker could not record binocular data.

Strengths of this binocular eye-tracking paradigm include interactivity and comfort to promote cooperation of pediatric patients, as well as objective, understandable and visualized results, providing the possibility for clinical application.

## Conclusion

Our findings suggested that amblyopic children have abnormal eye movement functions, with increased inaccuracy of sustained fixations and post-saccadic fixations. Timely amblyopia intervention can additionally restore the functions in children with resolved visual acuity. The present results highlight the value of binocular assessment of eye movements in amblyopia management.

## Supplementary Information

**Additional file 1: eTable 1.** Results of repeated measures.

**Additional file 2: eTable 2.** Subgroup analysis* of main outcome measures.

## Data Availability

The datasets used and/or analyzed during the current study available from the corresponding author on reasonable request.
